# Supplementary material for: Pyruvate Dehydrogenase Kinase 1 inhibition mediated oxidative phosphorylation enhancement in cartilage promotes osteoarthritis progression
Source: BMC Musculoskelet Disord. 2023 Jul 20;24:597. doi: 10.1186/s12891-023-06585-6 (PMC10357736; doi:10.1186/s12891-023-06585-6)
Supplement: Supplementary file 1 — Additional file 1. [file 12891_2023_6585_MOESM1_ESM.zip › BP.Gsea.1653623667859/GOBP_POSITIVE_REGULATION_OF_MIRNA_TRANSCRIPTION.html]

Details for gene set GOBP\_POSITIVE\_REGULATION\_OF\_MIRNA\_TRANSCRIPTION[GSEA]

|  || Dataset | 55457\_collapsed\_to\_symbols.cls.cls #OA\_versus\_normal.cls.cls #OA\_versus\_normal\_repos |
| Phenotype | cls.cls#OA\_versus\_normal\_repos |
| Upregulated in class | normal |
| GeneSet | GOBP\_POSITIVE\_REGULATION\_OF\_MIRNA\_TRANSCRIPTION |
| Enrichment Score (ES) | -0.54288167 |
| Normalized Enrichment Score (NES) | -2.0120215 |
| Nominal p-value | 0.0 |
| FDR q-value | 0.02677723 |
| FWER p-Value | 0.231 |
Table: GSEA Results Summary

  

Fig 1: Enrichment plot: GOBP\_POSITIVE\_REGULATION\_OF\_MIRNA\_TRANSCRIPTION      
 Profile of the Running ES Score & Positions of GeneSet Members on the Rank Ordered List

  

| SYMBOL | TITLE | RANK IN GENE LIST | RANK METRIC SCORE | RUNNING ES | CORE ENRICHMENT || 1 | NR3C1 | nuclear receptor subfamily 3 group C member 1 [Source:HGNC Symbol;Acc:HGNC:7978] | 674 | 0.599 | -0.0151 | No |
| 2 | TNF | tumor necrosis factor [Source:HGNC Symbol;Acc:HGNC:11892] | 2098 | 0.350 | -0.1040 | No |
| 3 | TGFB1 | transforming growth factor beta 1 [Source:HGNC Symbol;Acc:HGNC:11766] | 2490 | 0.303 | -0.1155 | No |
| 4 | BMPR1A | bone morphogenetic protein receptor type 1A [Source:HGNC Symbol;Acc:HGNC:1076] | 2745 | 0.275 | -0.1180 | No |
| 5 | SMAD6 | SMAD family member 6 [Source:HGNC Symbol;Acc:HGNC:6772] | 3460 | 0.206 | -0.1608 | No |
| 6 | RELA | "RELA proto-oncogene, NF-kB subunit [Source:HGNC Symbol;Acc:HGNC:9955]" | 3942 | 0.161 | -0.1881 | No |
| 7 | SMAD3 | SMAD family member 3 [Source:HGNC Symbol;Acc:HGNC:6769] | 4061 | 0.150 | -0.1880 | No |
| 8 | NGFR | nerve growth factor receptor [Source:HGNC Symbol;Acc:HGNC:7809] | 4156 | 0.142 | -0.1864 | No |
| 9 | SREBF1 | sterol regulatory element binding transcription factor 1 [Source:HGNC Symbol;Acc:HGNC:11289] | 4729 | 0.093 | -0.2251 | No |
| 10 | NR1H2 | nuclear receptor subfamily 1 group H member 2 [Source:HGNC Symbol;Acc:HGNC:7965] | 5413 | 0.039 | -0.2758 | No |
| 11 | WT1 | WT1 transcription factor [Source:HGNC Symbol;Acc:HGNC:12796] | 5452 | 0.037 | -0.2765 | No |
| 12 | PRL | prolactin [Source:HGNC Symbol;Acc:HGNC:9445] | 5490 | 0.033 | -0.2773 | No |
| 13 | TP53 | tumor protein p53 [Source:HGNC Symbol;Acc:HGNC:11998] | 5511 | 0.032 | -0.2769 | No |
| 14 | TERT | telomerase reverse transcriptase [Source:HGNC Symbol;Acc:HGNC:11730] | 5773 | 0.010 | -0.2965 | No |
| 15 | PPARG | peroxisome proliferator activated receptor gamma [Source:HGNC Symbol;Acc:HGNC:9236] | 6006 | -0.005 | -0.3143 | No |
| 16 | TGFB2 | transforming growth factor beta 2 [Source:HGNC Symbol;Acc:HGNC:11768] | 6985 | -0.079 | -0.3855 | No |
| 17 | FGF2 | fibroblast growth factor 2 [Source:HGNC Symbol;Acc:HGNC:3676] | 7183 | -0.095 | -0.3949 | No |
| 18 | STAT3 | signal transducer and activator of transcription 3 [Source:HGNC Symbol;Acc:HGNC:11364] | 8453 | -0.190 | -0.4817 | No |
| 19 | IL10 | interleukin 10 [Source:HGNC Symbol;Acc:HGNC:5962] | 8855 | -0.221 | -0.4991 | No |
| 20 | SPI1 | Spi-1 proto-oncogene [Source:HGNC Symbol;Acc:HGNC:11241] | 9012 | -0.232 | -0.4968 | No |
| 21 | SMAD4 | SMAD family member 4 [Source:HGNC Symbol;Acc:HGNC:6770] | 9035 | -0.235 | -0.4839 | No |
| 22 | ETS1 | "ETS proto-oncogene 1, transcription factor [Source:HGNC Symbol;Acc:HGNC:3488]" | 9795 | -0.301 | -0.5241 | Yes |
| 23 | SMAD1 | SMAD family member 1 [Source:HGNC Symbol;Acc:HGNC:6767] | 9905 | -0.312 | -0.5131 | Yes |
| 24 | SMARCA4 | "SWI/SNF related, matrix associated, actin dependent regulator of chromatin, subfamily a, member 4 [Source:HGNC Symbol;Acc:HGNC:11100]" | 10009 | -0.322 | -0.5011 | Yes |
| 25 | SREBF2 | sterol regulatory element binding transcription factor 2 [Source:HGNC Symbol;Acc:HGNC:11290] | 10070 | -0.328 | -0.4853 | Yes |
| 26 | FOS | "Fos proto-oncogene, AP-1 transcription factor subunit [Source:HGNC Symbol;Acc:HGNC:3796]" | 10640 | -0.387 | -0.5055 | Yes |
| 27 | GNL3 | G protein nucleolar 3 [Source:HGNC Symbol;Acc:HGNC:29931] | 10907 | -0.418 | -0.5001 | Yes |
| 28 | BMP2 | bone morphogenetic protein 2 [Source:HGNC Symbol;Acc:HGNC:1069] | 10919 | -0.420 | -0.4748 | Yes |
| 29 | GATA2 | GATA binding protein 2 [Source:HGNC Symbol;Acc:HGNC:4171] | 11058 | -0.439 | -0.4582 | Yes |
| 30 | FOSL1 | "FOS like 1, AP-1 transcription factor subunit [Source:HGNC Symbol;Acc:HGNC:13718]" | 11222 | -0.460 | -0.4422 | Yes |
| 31 | FOXO3 | forkhead box O3 [Source:HGNC Symbol;Acc:HGNC:3821] | 11305 | -0.472 | -0.4192 | Yes |
| 32 | TEAD1 | TEA domain transcription factor 1 [Source:HGNC Symbol;Acc:HGNC:11714] | 11379 | -0.483 | -0.3948 | Yes |
| 33 | MRTFA | myocardin related transcription factor A [Source:HGNC Symbol;Acc:HGNC:14334] | 11613 | -0.518 | -0.3806 | Yes |
| 34 | MRTFB | myocardin related transcription factor B [Source:HGNC Symbol;Acc:HGNC:29819] | 11668 | -0.527 | -0.3520 | Yes |
| 35 | HIF1A | hypoxia inducible factor 1 subunit alpha [Source:HGNC Symbol;Acc:HGNC:4910] | 11833 | -0.556 | -0.3301 | Yes |
| 36 | EGR1 | early growth response 1 [Source:HGNC Symbol;Acc:HGNC:3238] | 12052 | -0.607 | -0.3093 | Yes |
| 37 | GATA3 | GATA binding protein 3 [Source:HGNC Symbol;Acc:HGNC:4172] | 12276 | -0.670 | -0.2849 | Yes |
| 38 | PDGFB | platelet derived growth factor subunit B [Source:HGNC Symbol;Acc:HGNC:8800] | 12389 | -0.704 | -0.2497 | Yes |
| 39 | SRF | serum response factor [Source:HGNC Symbol;Acc:HGNC:11291] | 12549 | -0.773 | -0.2139 | Yes |
| 40 | KLF5 | Kruppel like factor 5 [Source:HGNC Symbol;Acc:HGNC:6349] | 12672 | -0.845 | -0.1708 | Yes |
| 41 | MYC | "MYC proto-oncogene, bHLH transcription factor [Source:HGNC Symbol;Acc:HGNC:7553]" | 12801 | -0.970 | -0.1203 | Yes |
| 42 | JUN | "Jun proto-oncogene, AP-1 transcription factor subunit [Source:HGNC Symbol;Acc:HGNC:6204]" | 12813 | -0.994 | -0.0592 | Yes |
| 43 | KLF4 | Kruppel like factor 4 [Source:HGNC Symbol;Acc:HGNC:6348] | 12848 | -1.058 | 0.0040 | Yes |
Table: GSEA details [plain text format]

  

Fig 2: GOBP\_POSITIVE\_REGULATION\_OF\_MIRNA\_TRANSCRIPTION      
 Blue-Pink O' Gram in the Space of the Analyzed GeneSet

  

Fig 3: GOBP\_POSITIVE\_REGULATION\_OF\_MIRNA\_TRANSCRIPTION: Random ES distribution      
 Gene set null distribution of ES for **GOBP\_POSITIVE\_REGULATION\_OF\_MIRNA\_TRANSCRIPTION**
